# Treatment of Obesity and Fatty Degeneration

**Published:** 1893-09

**Authors:** G. H. Thompson

**Affiliations:** St. Louis, Mo.; Prof. Materia Medica St. Louis College of Physicians and Surgeons


### TREATMENT OF OBESITY AND FATTY DEGENERATION.

BY G. H. THOMPSON, A. M., M. D., PROF. MATERIA MEDICA ST. LOUIS COLLEGE OF PHYSICIANS AND SURGEONS.

It is not necessary to enter into a discussion of what constitutes fatty degeneration, suffice it to say that this condition generally co-exists with a tendency to general obesity or results from the abuse of alcoholics. Those having a tendency to general obesity, are usually partial also to diets rich in sugar and starches, and small amounts of carbo hydrates added to such diet, greatly favors the deposition of fat. The hereditary tendency to accumulation of fat, in my experience, usually manifests itself in males at the age of thirty, sometimes earlier, sometimes later. In females usually at about twenty-eight. As long as fat serves its functions only—that of adding rotundity to the form and serving as food supply—in times of sickness there is no necessity of seeking to eliminate it. It is, when it accumulates persistently, causing distress by its weight and interference with locomotion, or when internal viscera become so degenerated by substitution of fat for normal organic tissue, that distress is caused or life threatened, that we should seek to correct its effects.

The members of the medical profession doubtless have their own ideas as to whether relief should be given on purely cosmetic grounds, or not, and it is not my intention to discuss this subject from such a standpoint. The question is, how can necessary relief be afforded? In endeavoring to throw some light on this question, I take occasion to report a few cases:

Case 1.—Mrs. E., aged 28, weight 139 pounds, height five feet. Complained that she suffered from heat. For two years she has been unable to stand the moderate summer elevations of temperature without great inconvenience. Perspired so freely that she was obliged to remain indoors most of the time between June and September on account of ruining her clothes with perspiration. No female or other trouble was present, but she seemed to have a superabundance of adipose tissue generally distributed, especially about the chest and abdomen. Habits regular, bowels and courses likewise. Concluded to see the effect of reducing her weight, and for this purpose, after trying Fucus Vesiculus in the dose of one to two drams of the fluid extract three times a day, with some slight benefit, I determined to try Phytolacca Decandra, which has been recommended by Dr. M. M. Griffith, as a potent measure in diminishing obesity. The preparation used was Phytoline, (Walker,) a remedy prepared from the active principle of the Phytholacca Berries, after being somewhat frost-bitten. The dose first used was gtt. x v four times a day. The patient used two bottles, after which she reported herself feeling very much improved, perspiration lessened, weight 128 pounds, appetite about the same, regular bowels and courses. I could find no bad effects from the remedy.

Case 2.—H. W. M., aged 33, weight 160 pounds, height five feet, six inches. Complained of præcordial distress, difficult breathing, occasional attacks of vertigo, heart-beat feeble, irregular and slow, sometimes rapid, anaemia, weakness in the legs, which were not very muscular. Patient was addicted to alcoholics, suffered consequently from dyspepsia and atony of the bowels. Diagnosis : fatty degeneration of the heart due to alcoholism. Stopped his alcoholics, administered stomachic tonic of quinine, strychnine and capsicum, and gave Phytolacca (Phytoline-Walker) in the dose of gtt. x, six times a day before and after each meal. In three weeks' time there was a notable improvement in every respect. Weight had decreased five pounds, heart-beat fuller and more regular, præcordial distress and difficult breathing ceased altogether, digestion improved, appetite likewise. Patient was on the road to recovery when persistent exposure to extreme cold brought on pneumonia, from which he died after five days' illness.

In these two cases there was no advice given as to diet, except the withdrawal of alcoholics in the last case, it also being remembered that alcoholics antagonize the action of Phytolacca.

Case 3.—Mr. N., aged 54, weight 240 pounds, height five feet, ten inches. Complained of eczema of the legs and left side. Inspection showed in the left hypochondrium, a large circumscribed ulceration about two inches in diameter, surrounded by inflamed vesicular-area ; the legs showed similar ulceration in the skin. Patient perspired freely, almost to a point of hyperidrosis. During cold weather patient was not troubled except from difficult locomotion and occasional attacks of rheumatism. Examination of urine showed no sugar. Appetite fair, drank considerable beer, bowels regular. Astringent salves and lotions cured temporarily. Diagnosed eczema, due to maceration. Placed patient on Phytolacca (Phytoline Walker's) gtt. xxv., before and after each meal. In two weeks patient lost 10 pounds, had somewhat less appetite, less perspiration, sores took on a healthier condition, and after continuing the treatment about two months and a half, patient felt as well as ever, and tipped the beam at 200. Since then the patient has gained but little, if any, perspires normally and has no return of his eczema and no recurrence of rheumatism. How long this condition will last, time alone can tell.

This last case was one, especially calling for some fat reducing agent, and the checking of perspiration. In this case bread and potatoes were prohibited, likewise other form of starchy foods ; beer was reduced in quantity two-thirds. These measures materially increased the fat reducing properties of the Phytoline.

The next question is, how does Phytoline cause the reduction of fat ? This, I am at present, unable to say. I have, however, noticed that the faeces seemed to be considerably more rich in fatty materials than is normal, which condition cannot be attributed to indigestion, as in all other respects digestion was normal. Perspiration and urine was apparently unchanged by the action of the drug.

Officinal preparation of the root have been used with little or no result, except to cause continued nausea, vomiting and diarrhœa. Phytoline does not cause nausea in the ordinary dose, and though slight laxative effects have been observed from it, I have never seen a pronounced case of diarrhœa. The appetite is sometimes slightly diminished chiefly in the morning. It seems to me to be specially indicated in all diseases characterized by fatty degeneration of internal viscera, especially of the heart and liver. Those who chose to use it for its cosmetic effects in reducing fat, will also find in it a serviceable adjunct to properly restricted diet and exercise.

